# Editorial: Research into talent development in youth sports

**DOI:** 10.3389/fspor.2023.1257643

**Published:** 2023-08-10

**Authors:** Arne Güllich, Carlos E. Gonçalves, Humberto M. Carvalho

**Affiliations:** ^1^ Department of Sports Science, RPTU Kaiserslautern, Kaiserslautern, Germany; ^2^ School of Sports, Federal University of Santa Catarina, Florianopolis, Brazil

**Keywords:** youth sports, talent identification, talent development, review, theory, research programme

**Editorial on the Research Topic** Research into talent development in youth sports

What explains exceptional performance? This is the subject of one of the oldest lines of scientific research (1, 2). Traditionally, one community of thought (3) has emphasised the importance of inborn “natural abilities” and initial performance level [giftedness approach, e.g., (1, 4)], yet acknowledging the relevance of a long-term practice process. Another community of thought has emphasised the importance of the practice process [environmentalist approach, e.g., (2, 5)], yet acknowledging the relevance of physical attributes and early performance.

In sports, dedicated research into talent development has begun in the 1960s [e.g., (6, 7)] and has then continuously grown, in parallel with the expansion, popularity, and commercialisation of the sport industry. Today, many national sport systems around the world have established talent promotion programmes at local, regional, and national levels. Talent promotion is considered a critical building block of athletes' pathway towards athletic excellence and the “global sporting arms race” has incited nations to make expanding strategic investments in talent promotion programmes.

**Figure 1:**
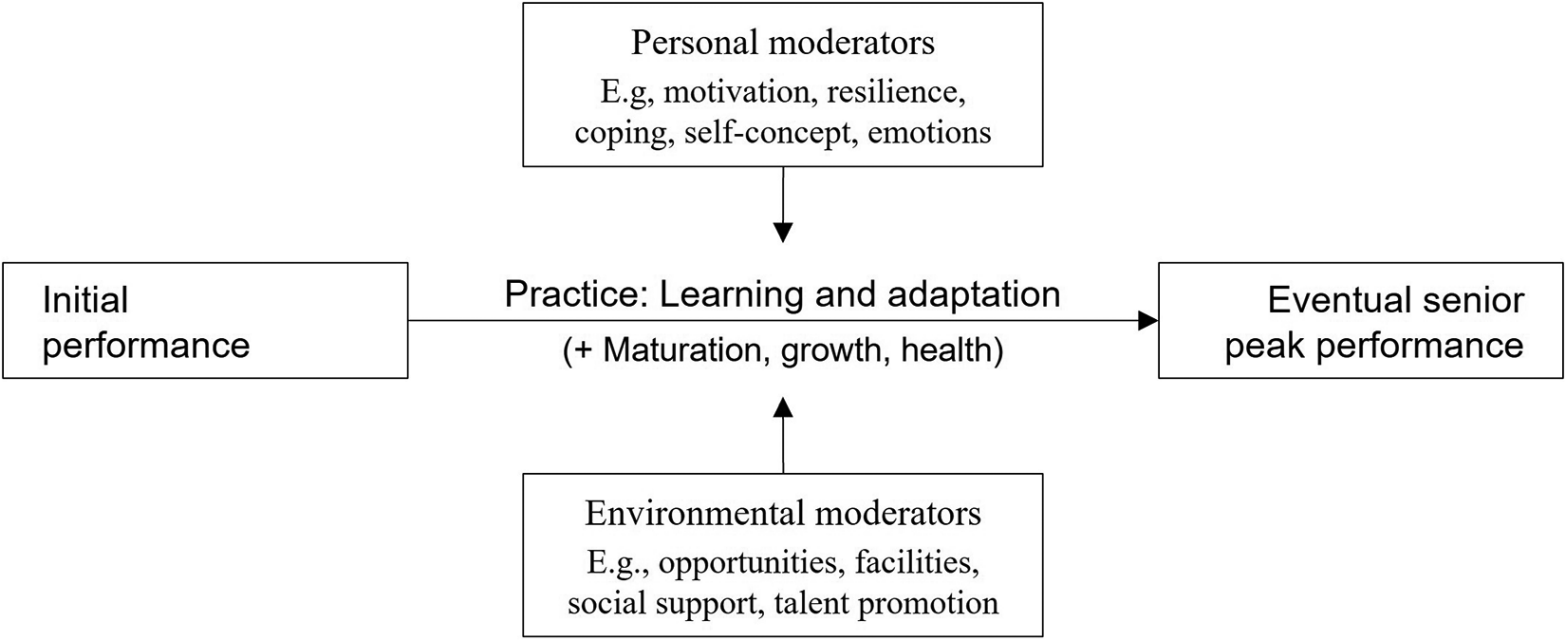
Basic model of talent development.

Although theoretical approaches to talent development partly vary, there is large consensus that every youth athlete has some initial level of performance. Their subsequent performance development is driven via a multi-year practice process (typically composed of drill-like exercise forms, playing forms, and competitions), which is moderated by personal and environmental factors. This practice process eventually leads to their senior peak performance (Figure 1).

This book assembles 13 reviews of available research on many of these subjects. Four chapters focus on characteristics of the practice process itself (Figure 1). Araújo et al. first explain how “talent” is socially defined. Then, based on an ecological-dynamics rationale, they discuss talent development as a socialisation process transforming ubiquitous skills into specialised skills via exploration, stabilisation, and calibration of the performer-environment coupling. Larkin et al. review existing research into the micro-structure of youth athletes' practice sessions, especially the allocation of training vs. playing forms. They also discuss coaches' behaviours employed to foster athletes' learning and performance. Güllich et al. synthesise the available empirical evidence on participation patterns of higher- and lower-performing athletes. Predictors of early junior performance and of long-term senior performance (i.e., in the highest, open-age category) are opposite in five regards: starting age, amounts of main-sport and other-sports practice, age to enter talent promotion programmes, and age to reach defined performance “milestones.” Peters et al. analyse the literature specifically addressing girls' and women's participation variables. The participation patterns of many successful female athletes deviate from popular theoretical hypotheses such as Ericsson et al.'s (5) “deliberate practice” framework and Côté et al.'s (8) “Developmental Model of Sport Participation.”

Three chapters discuss several potential risks associated with talent development, including those of selection biases. Carvalho and Gonçalves illustrate how youth athletes' varying timing of biological maturation (puberty, growth spurt) and relative age within a birth-year cause specific biases in talent selection. This leads to increased risks of false-positive and false-negative selection decisions, given that both the biological-age effect and relative-age effect diminish or may even be reversed by adulthood. Wik describes injuries in talent development, exemplified by youth soccer. He explains how players' age, biological maturity, and growth affect the prevalence, types, and locations of injuries, highlighting particular vulnerability of youth athletes' growth plates and apophyses. Soares and Carvalho discuss fundamental issues associated with previous research into dropout of young talents. Dropout studies have typically addressed sport-specific, not general sport dropout; considered unselected, not talent development populations; and definitions of dropout have varied. In consequence, substantiated knowledge about the actual prevalence and factors of dropout from talent development pathways is still meagre.

Five chapters address several moderators of the process of practice and performance development (Figure 1). Weissensteiner discusses international trends in the historical development of national talent promotion systems, illustrated by the GDR, Australia, and the UK. Employing historical analyses, she works out the commonalities and particularities of three extremely successful talent promotion systems, and key learnings each of them obtained from the previous one. Hancock et al. review the state of research into the geography of talent development. Athletes born in places with medium population size and density typically have increased success probabilities. The authors also acknowledge that birthplace effects vary across sports, countries, and sexes; definitions of “medium” population size and density differ between countries; and athletes' birthplace and development place(s) may not be identical. Taking a holistic ecological approach, Henriksen and Stambulova conceptualise the athletic career as a journey through varying athletic and non-athletic social environments. They summarise qualitative investigations of successful environments and highlight shared features regarding organisational structure and culture that have been perceived to foster athletes’ performance, wellbeing, and personal development. Quinaud et al. address the combined athletic and academic development of youth athletes, labelled “dual career.” Combining athletic and academic engagement implies competing time demands from sport and education. Considering position and policy papers, the authors call for clear definitions of guidelines, resources, roles, and responsibilities in the establishment of dual-career support programmes. Dehghansai et al. show that traditional talent development models are only partly applicable, at best, to Paralympic sports. Athletes' development differs between congenital vs. acquired impairment and across ages of acquiring an impairment. Furthermore, types and severity of impairments require varying resources in terms of equipment and coaching, and it is difficult to establish classifications that ensure fair competition systems. In conclusion, Paralympic talent development requires especial dedication, flexibility, creativity, and resources.

Finally, Baker et al. advocate for embedding talent development models and research in multidimensional lifespan development models and research. The authors highlight the complexity of athletes' development within and between competitive and recreational participation and discuss challenges associated with that research.

Generally, an overarching research question concerning all the potential factors of talent development is: To what extent do individual differences in childhood/adolescent factors predict individual differences in later senior performance? Given that youth athletes, parents, and coaches seek to expand athletes' benefits (e.g., enjoyment, performance, prestige) while controlling and limiting their risks (e.g., injuries, burnout, dropout) and costs (especially opportunity costs, i.e., the lost benefit of foregone other activities such as time with family, friends, academics; declining academic achievements; declining response to training with growing previous training amounts; reduced psychosocial wellbeing), that research question can be further specified: What childhood/adolescent factors facilitate long-term senior performance, and *at what risks and costs*?.

Researchers elaborate theories that are then evaluated based on two truth values: logical consistency and empirical correspondence, where their empirical content constitutes their potential falsifiers (9). I.e., researchers propose systems of hypotheses and nature disposes of their truth or falsity (10). For many potential factors in talent development, multi-year experimental manipulation is difficult, if not impossible, for example: training volume and methods, parental and peer support, athletes' psychological characteristics, health, or psychosocial wellbeing. The methods of choice are therefore typically multi-year longitudinal quasi-experiments using prospective and retrospective designs while seeking to control for potential confounds.

There is a group of factors for which ample childhood/adolescent data of (later) senior athletes are available. For example, data on competitive performance development (11), relative age (Carvalho & Gonçalves), and birthplace (Hancock et al.) can typically be gathered from public records. Biological maturation (Carvalho & Gonçalves) and childhood/adolescent motor test scores are sometimes available from past routine monitoring procedures (12). Furthermore, athletes can reliably recall childhood/adolescent participation variables and involvement in talent promotion programmes (Güllich et al.; Peters et al.; Quinaud et al.; Dehghansai et al.) in retrospective interviews or questionnaires. This has led to a broad body of evidence on effects of these childhood/adolescent predictors on long-term senior performance across wide ranges of sports, performance levels, and countries.

Research into another group of potential factors is more difficult. For example, investigating the extent to which higher- and lower-performing senior athletes differed in earlier childhood/adolescent factors such as: 1. their microstructure of practice (Larkin et al.); 2. correspondence of their practice to principles of ecological dynamics (Araújo et al.); 3. psychological characteristics [e.g., (13, 14)]; 4. characteristics of athletes' social environment (Henriksen & Stambulova); or 5. support measures applied in talent promotion and dual-career programmes (Güllich et al.; Quinaud et al.). These variables are usually not available from public records or past routine monitoring procedures and senior athletes cannot reliably reconstruct them from their early years. This difficulty is perhaps one of the reasons why for these potential predictors, there is a broad body of theoretical hypotheses, normative assumptions, descriptive studies of youth athletes, and investigations of short-term effects on early junior performance. In contrast, evidence on effects of individual childhood/adolescence differences in these factors on long-term individual differences in senior performance is lacking.

However, we cannot infer predictors of senior performance by extrapolating findings from junior athletes because 1. successful juniors and successful seniors are largely two disparate populations (11) and 2. predictors of early junior performance vs. long-term senior performance are different and partly opposite (Güllich et al.; Carvalho & Gonçalves). Likewise, although the goal is to expand athletes' benefits while limiting their risks and costs (see above), there is only scarce empirical evidence, if any, concerning childhood/adolescent predictors of adult high performance *combined* with other outcomes in adulthood such as psychosocial wellbeing (Henriksen & Stambulova), health (Wik), academic/vocational achievement (Quinaud et al.), or prolonged sport engagement (Soares & Carvalho; Baker et al.).

The chapters in this book suggest several clear implications for future research.

1. The process of talent development is complex and multi-factorial, calling for more multi-theoretical approaches and multivariate analyses of interactions between factors. In addition, associations between several childhood/adolescent predictors and senior-age outcomes are likely non-linear rather than linear, while organised in multi-level structures. For example, based on the available evidence, several relationships are presumably better reflected by parabolic (e.g., earlier cumulative practice amount and later performance), saturation (e.g., earlier motivation and later performance), or threshold patterns (e.g., earlier cumulative physical load and later overuse injury). These plausibility assumptions call for multivariate non-linear analyses and advanced modelling.
2. We should seek to expand the empirical evidence on long-term effects of several hypothesised childhood/adolescent factors that are under-researched to date: E.g., early talent indicators, talent selection criteria, microstructure of practice, its correspondence to principles of ecological dynamics, psychological characteristics, social environment, parental and peer support, and support measures applied to participants in talent promotion and dual-career programmes. This implies investigating the research question: To what extent had (a) higher- vs. lower-performing senior athletes with (b) better vs. poorer wellbeing, health, or academic/vocational achievement differed in these factors during childhood/adolescence.
3. Given that (1) the goal is to expand the athlete's benefits while limiting their risks and costs, while (2) effects of childhood/adolescent factors may vary and even be opposite regarding short-term and long-term outcomes, the economic concepts of efficiency of practice—performance improvement per invested practice amount—and sustainability are paramount. They apply to research into youth athletes' participation patterns, microstructure of practice, ecological dynamics, coaching, talent promotion programmes, dual-career support, athlete services, and youth sport programmes in general, and lead to three critical research questions (Güllich et al.): (a) What short- and long-term, material and immaterial benefits, risks, and costs does a programme (or do different programmes) yield? (b) What objective and subjective value does each of the benefits, risks, and costs have? (c) What is the eventual ratio of the summed value of all benefits relative to the summed value of all risks and costs yielded by a programme (or by different programmes)?

This research will advance our understanding of long-term talent development, foster our refinement of sound theories, provide the corresponding empirical evidence, and thereby facilitate evidence-based practice of talent development.
